# A Third Surgically Managed Ectopic Pregnancy after Two Salpingectomies Involving the Opposite Tube

**DOI:** 10.1155/2017/1653529

**Published:** 2017-01-02

**Authors:** Naoyuki Iwahashi, Yoko Deguchi, Yuko Horiuchi, Kazuhiko Ino, Kenichi Furukawa

**Affiliations:** ^1^Department of Obstetrics and Gynecology, Hashimoto Municipal Hospital, Wakayama, Japan; ^2^Department of Obstetrics and Gynecology, Wakayama Medical University, Wakayama, Japan

## Abstract

Recurrent ectopic pregnancy in a remnant fallopian tube after ipsilateral salpingectomy is clinically rare. We report the extremely rare case of a third recurrent ectopic pregnancy after two previous salpingectomy procedures involving the opposite tube. A 26-year-old woman, gravida 3 para 0, experienced three ectopic pregnancies brought about by natural conception, all of which were treated surgically (right partial salpingectomy, right remnant tube resection, and left total salpingectomy). During the two salpingectomy procedures involving the right tube, the patency of the intact left tube was intraoperatively confirmed with indigo carmine. The most appropriate surgical intervention should be discussed when managing recurrent ectopic pregnancies. It might be necessary to perform total salpingectomy to reduce the risk of future recurrence on the remaining tube.

## 1. Introduction

Ectopic pregnancies account for 1-2% of all pregnancies, and the rupturing of ectopic pregnancies can cause massive bleeding and maternal mortality [1]. Although the risk of recurrent ectopic pregnancies increases after previous medical and surgical management, it is reduced after salpingectomy [2–4]. Recurrent ectopic pregnancy in a remnant tube after ipsilateral salpingectomy is rare, and less than 20 cases have been reported in the English literature [5–7]. In addition, there has only been one reported case of three surgically managed ipsilateral ectopic pregnancies [8]. We experienced a rare case in which a third ectopic pregnancy occurred in the intact tube after two salpingectomy procedures involving the opposite tube.

## 2. Case Presentation

A 26-year-old gravida 3, para 0 female was referred to our hospital at 6 + 5 weeks of gestation due to a suspicion of left tubal ectopic pregnancy. The patient's medical history included one miscarriage and two ectopic pregnancies in the right tube, which had been surgically treated with right partial salpingectomy 4 years ago and right remnant salpingectomy 2 years ago, respectively. During these two ipsilateral salpingectomy procedures, normal left tubal patency was confirmed by chromotubation using indigo carmine. All of the pregnancies were established via natural conception, and during the first ectopic pregnancy the patient was diagnosed with a Chlamydia trachomatis infection. On admission, she was free from abdominal pain and vaginal discharge. Transvaginal ultrasonography revealed the absence of an intrauterine pregnancy and the presence of a left tubal ectopic pregnancy (a fetus and a fetal heartbeat were detected). The patient exhibited a serum *β*-human chorionic gonadotropin (hCG) level of 4,000 international units/mL. We fully discussed the surgical options with the patient, whether to perform salpingectomy or salpingotomy, and she selected salpingectomy, even though this would result in the loss of her natural fertility, as she was concerned about the risk of a fourth ectopic pregnancy. Due to the appearance of marked peritoneal irritation, an emergent laparotomy was performed. We found massive hemoperitoneum, and a left tubal isthmus ectopic pregnancy. To prevent any further recurrence, we performed left complete salpingectomy based on the patient's wishes. The clinical diagnosis of tubal ectopic pregnancy was confirmed histologically. The patient's postoperative course was uneventful, and a serum *β*-hCG test produced a negative result at one postoperative month. The patient gave birth after in vitro fertilization without any problems.

## 3. Discussion

This report highlights an interesting clinical case of three surgically managed recurrent ectopic pregnancies brought about by natural conception, which were surgically treated with right partial salpingectomy, right resection of the remnant tube, and left total salpingectomy, respectively. The third tubal pregnancy occurred in the intact left tube, the patency of which was confirmed by chromotubation using indigo carmine during the previous two salpingectomy procedures.

To the best of our knowledge, this is the first report about a third surgically managed recurrent ectopic pregnancy in an intact tube after two salpingectomy procedures involving the opposite tube. Recurrent ectopic pregnancy in the remnant tube after ipsilateral salpingectomy is exceptionally rare [3–5]; thus, the frequency of serious complications in such cases remains unclear. Furthermore, there has only been one report about three consecutive surgically managed ipsilateral ectopic pregnancies [8]. Three hypotheses regarding the mechanisms responsible for recurrent ectopic pregnancies after ipsilateral salpingectomy have been proposed: contralateral fertilized egg migration across the endometrium to the remnant fallopian tube, contralateral fertilized egg trans-peritoneal passage through the contralateral intact fallopian tube, and recanalization of the tubal remnant [5, 8, 9]. In some cases of recurrent ectopic pregnancy brought about by natural conception after ipsilateral salpingectomy, it might be necessary to preserve the normal contralateral tube, as seen in our case. We confirmed the patency of the normal contralateral tube during the two previous ipsilateral salpingectomy procedures via intraoperative chromotubation. Even in cases involving natural conception, we should consider the risk of a third recurrent ectopic pregnancy in the normal tube.

The optimal management strategy for recurrent ectopic pregnancy was unclear. The risk of recurrent ectopic pregnancy is reported to be fourfold higher in cases involving previous medical or surgical management [10], and the risk of such pregnancies does not differ significantly between medically and surgically treated cases. Total and partial salpingectomy might be selected in cases in which the patient does not want any further children, but salpingotomy might be chosen to preserve future natural fertility, especially in primiparous women. In a large retrospective study, salpingotomy was found to be significantly better at preserving fertility than total salpingectomy, although total salpingectomy carries a lower risk of recurrent ectopic pregnancy [2, 3, 11]. Although complete tubal resection cannot prevent cornual pregnancy, it might reduce the risk of recurrent ectopic pregnancy in the remnant tube. In cases of recurrent ectopic pregnancy, the surgical management strategy should be fully discussed with patient and their family, and appropriate informed consent should be obtained. In the present case, partial salpingectomy was performed the first ectopic pregnancy, which might induce the second recurrent ectopic pregnancy treated by resection of remnant tube, followed by the third recurrent ectopic pregnancy with total salpingectomy.

We described the first reported case of a third surgically managed recurrent ectopic pregnancy in an intact tube after two salpingectomy procedures involving the opposite tube. Obstetricians should be aware of the possibility of such rare cases, and the available surgical interventions should be fully discussed with patients when managing recurrent ectopic pregnancies. To avoid the risk of future recurrence in the remnant tube, it might be necessary to perform total salpingectomy rather than partial salpingectomy.
